# Using mice from different breeding sites fails to improve replicability of results from single-laboratory studies

**DOI:** 10.1038/s41684-023-01307-w

**Published:** 2023-12-27

**Authors:** Ivana Jaric, Bernhard Voelkl, Irmgard Amrein, David P. Wolfer, Janja Novak, Carlotta Detotto, Ulrike Weber-Stadlbauer, Urs Meyer, Francesca Manuella, Isabelle M. Mansuy, Hanno Würbel

**Affiliations:** 1 Animal Welfare Division, Vetsuisse Faculty, University of Bern, Bern, Switzerland; 2 Institute of Anatomy, Division of Functional Neuroanatomy, University of Zürich, Zürich, Switzerland; 3 Department of Health Sciences and Technology, ETH Zürich, Zürich, Switzerland; 4 Central Animal Facilities, Experimental Animal Center, University of Bern, Bern, Switzerland; 5 Institute of Pharmacology and Toxicology, Vetsuisse Faculty and Center of Neuroscience Zürich, University of Zürich, Zürich, Switzerland; 6 Laboratory of Neuroepigenetics, Brain Research Institute, Medical Faculty, University of Zürich, Zürich, Switzerland; 7 Institute for Neuroscience, Department of Health Science and Technology, Swiss Federal Institute of Technology Zürich (ETHZ), Zurich, Switzerland; 8 Center for Neuroscience Zürich, University Zürich and ETHZ, Zürich, Switzerland

**Keywords:** Animal behaviour, Robustness

## Abstract

Theoretical and empirical evidence indicates that low external validity due to rigorous standardization of study populations is a cause of poor replicability in animal research. Here we report a multi-laboratory study aimed at investigating whether heterogenization of study populations by using animals from different breeding sites increases the replicability of results from single-laboratory studies. We used male C57BL/6J mice from six different breeding sites to test a standardized against a heterogenized (HET) study design in six independent replicate test laboratories. For the standardized design, each laboratory ordered mice from a single breeding site (each laboratory from a different one), while for the HET design, each laboratory ordered proportionate numbers of mice from the five remaining breeding sites. To test our hypothesis, we assessed 14 outcome variables, including body weight, behavioral measures obtained from a single session on an elevated plus maze, and clinical blood parameters. Both breeding site and test laboratory affected variation in outcome variables, but the effect of test laboratory was more pronounced for most outcome variables. Moreover, heterogenization of study populations by breeding site (HET) did not reduce variation in outcome variables between test laboratories, which was most likely due to the fact that breeding site had only little effect on variation in outcome variables, thereby limiting the scope for HET to reduce between-lab variation. We conclude that heterogenization of study populations by breeding site has limited capacity for improving the replicability of results from single-laboratory animal studies.

## Abstract (web summary)

Poor replicability in animal research can be a result of low external validity driven by rigorous standardization of study populations. This study investigates how heterogenization of study populations by using mice from different breeding sites might affect the replicability of animal studies conducted in a single facility. The findings suggest that heterogenization by breeding site has limited capacity to improve replicability in animal research.

## Main

Experimental animal research is usually conducted using animals of the same genotype (inbred or mutant strains) reared and housed under almost identical conditions^1^. Such rigorous genetic and environmental standardization can produce study-specific results that lack external validity^2–4^, thereby causing poor replicability^5–7^. Theoretical and empirical evidence indicates that systematic heterogenization of study populations, rather than standardization, is needed to improve external validity and replicability^6,8–11^. However, previous studies indicate that simple forms of heterogenization (for example, varying cage size, group size, environmental enrichment or including multiple experimenters) are not effective enough to attenuate the large heterogeneity that normally exists between independent replicate studies^8,12,13^. Therefore, there is a need for more effective ways of heterogenizing study cohorts within single-laboratory studies to generate results that are replicable across independent laboratories.

We recently found that common environmental differences between animal facilities produce facility-specific phenotypes in mice, from the molecular to the behavioral level^14^. These findings suggest that the animals’ environmental background may serve as an effective heterogenization factor^14^. In this Article, we therefore tested whether systematic heterogenization of study populations, by using mice from different breeding sites to introduce the genetic and environmental variation that normally exists between independent study populations, would increase the external validity of the results sufficiently to guarantee replicability.

**Fig. 1:**
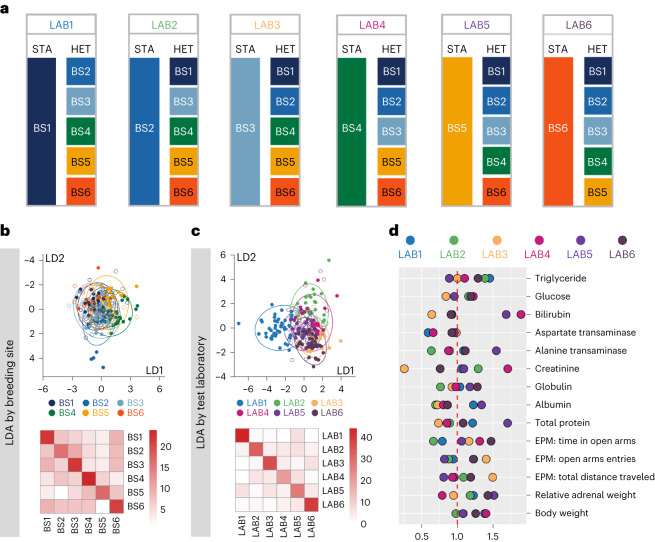
Effects of heterogenization on phenotypes. **a**, Schematic illustration of the multi-laboratory study design. **b**,**c**, The phenotype of mice was shaped by both breeding site (**b**) and test laboratory (**c**). In the LDA plots, color indicates breeder (**b**) and test laboratory (**c**). Filled dots represent a correct classification based on LDA, empty dots represent mismatches. **d**, Ratio of standard deviations of HET over STA cohorts for each outcome variable and each laboratory. A ratio >1 indicates that variation was larger in the HET compared to the STA cohort. BS, breeding site; LAB, test laboratory; LD, linear discriminant.

We used male C57BL/6J mice as a worked example and conducted a multi-laboratory study, with the same experiment conducted independently in six different laboratories by the same experimenter using the same test equipment. Each laboratory simultaneously employed both a standardized (STA) and a heterogenized (HET) study design (Fig. 1a). For STA, each laboratory ordered all mice (*n* = 24) in one cohort from one of six breeding sites (each laboratory from a different breeding site) to mimic the real-world situation of researchers independently ordering mice from a breeding site of their choice. By contrast, for HET, each laboratory ordered proportionate numbers of mice from the other five breeding sites (*n* = 30; 6 per lab; excluding the breeding site of the mice used in the STA design) to heterogenize the study population by the phenotypic variation that exists between mice from independent breeding sites (Fig. 1a). To test our hypothesis, we assessed 14 outcome variables, including body weight, behavioral measures obtained from a single session on an elevated plus maze (EPM), and clinical blood parameters. To eliminate potential sources of variation introduced by different experimenters and local test equipment, all mice underwent testing by the same experimenter using identical test equipment.

This approach allowed us to: (1) disentangle variance components originating from breeding site (combined effects of the genetic and environmental background) and test laboratory (where the experimental part of the study was performed); (2) test whether systematic heterogenization of study populations by using animals from different breeding sites increased the variance of the HET cohort compared to the STA cohort; and (3) evaluate the effectiveness of the HET design in improving replicability by meta-analyses for each outcome measure.

We found that both breeding site and test laboratory affected variation in outcome variables, but the effect of the test laboratory was much stronger than that of breeding site, despite the standardization of test equipment and test procedures. Since breeding site did not have a strong effect on variation in outcome variables, heterogenization by breeding site was not effective in improving the replicability of the results across laboratories.

## Results

We obtained samples of 14 outcome variables from 308 mice, resulting in 4,283 outcome measures after accounting for missing values (Extended Data Table 1 and Methods). Both breeding site and test laboratory, as well as their interaction, had significant effects on variation in outcome variables (multivariate analysis of variance (MANOVA), Extended Data Table 2). Whereas laboratory explained 11.2% of the multivariate variance, breeding site accounted for only 4.0%, and 11.4% were due to the interaction between breeding site and test laboratory (*η*^2^ estimates based on Pillai statistic). In a linear discriminant analysis (LDA) by breeding site, the first two discriminant functions explained 68% of the total variation (Extended Data Table 3), with LDA correctly predicting breeding site in 41% of cases (Fig. 1b) compared to 17% expected by chance. However, in an LDA by test laboratory, the first two discriminant functions accounted for even 79% of the total variation and correctly predicted the test laboratory in 66% of cases (Fig. 1c and Extended Data Table 4).

Post-hoc analyses of variance for individual outcome measures with breeding site and test laboratory as fixed effects and cage as random effect confirmed that breeding site and test laboratory together explained on average 26% of the total variation (range 10–43%). Thus, both the origin of the animals (breeding site) and the test conditions (test laboratory) affected the outcome measures, but test laboratory had a stronger effect than breeding site. Indeed, in 13 out of 14 outcome variables, test laboratory accounted for more of the variance than breeding site (Extended Data Table 5). We note that in some cases the post-hoc models produced a singular or boundary fit, which means that the covariance matrix may not be estimated correctly. The outcomes of those analysis of variance (ANOVA) models should thus be interpreted carefully.

To assess whether heterogenization by breeding site increased within-laboratory variance at the expense of between-laboratory variance, we compared the variance of each outcome variable between STA and HET cohorts for each laboratory. Variance was larger in HET cohorts than in STA cohorts in 45 cases but smaller in 39 cases, although differences were generally small. A statistically significant difference between HET and STA cohorts was detected in only 1 out of the 84 contrasts (Levene tests for equal variances, Extended Data Table 6), which is even below the expected rate of false positive findings (4.2), given *α* = 0.05. After adjusting the *α*-level threshold for multiple testing using a Bonferroni correction (*α*′ = 5.9 × 10^−4^), not a single statistically significant difference was detected. When combining outcome variables from the six test laboratories to obtain a single measure for each outcome variable, we did not find a significant difference between variances in the STA and HET cohorts for any of the 14 outcome measures (Extended Data Table 7). Thus, both at the level of individual contrasts and at the level of outcome variables, we found no evidence that variance was larger in HET cohorts.

Since each HET cohort contained animals from five of the six breeding sites used for STA cohorts, we expected lower between-laboratory variance in HET compared to STA cohorts. However, we found equal proportions of variance for test laboratory in HET cohorts (15.3%, range 4.9–40.4%) and STA cohorts (15.3%, range 3.7–40.3%). For 8 out of 14 outcome variables, between-laboratory variation was larger in STA cohorts, but for the other 6, it was larger in HET cohorts (Fig. 1d). We conclude that heterogenization of study populations by using mice from different breeding sites did not reduce between-laboratory variation.

**Fig. 2:**
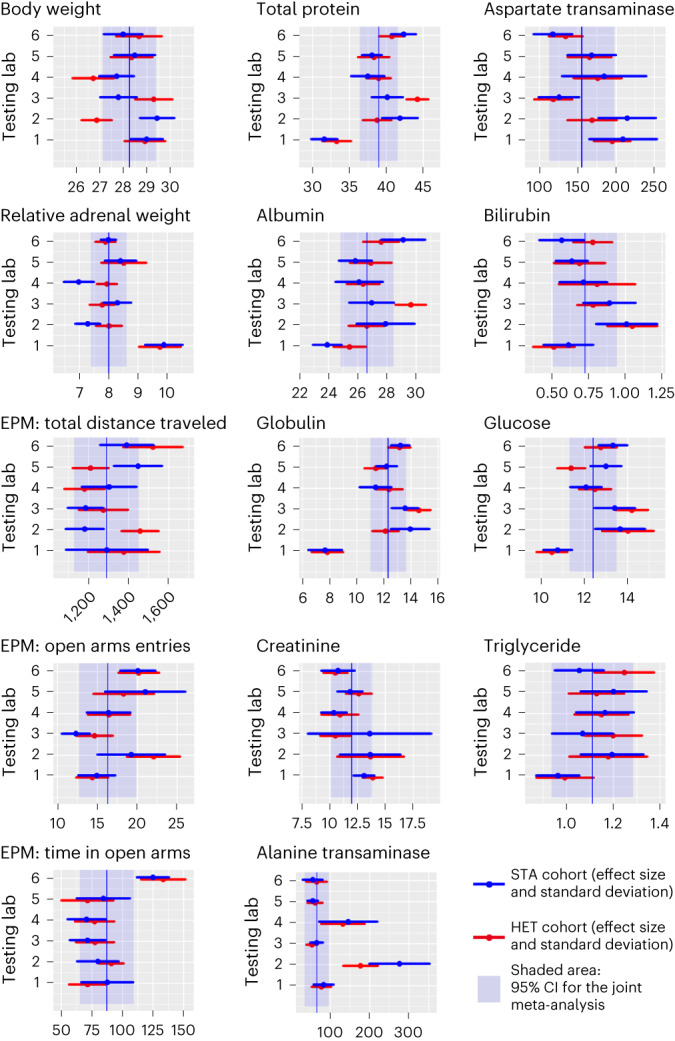
Meta-analysis for each outcome variable depending on study design. Heterogenization of study populations by breeding site (HET) did not improve replicability compared to a STA study design.

Finally, treating the results from the six test laboratories as replicate studies, we conducted meta-analyses for each outcome variable for both the HET and STA study designs. We predicted that study means deviate less from the meta-analytic mean in HET cohorts than in STA cohorts. However, random effects meta-analyses showed similar results for both HET and STA cohorts (Fig. 2 and Supplementary Fig. 1). A mixed-effect model with cohort as fixed factor and outcome measure and test laboratory as random factors suggests that for all outcome variables, study design (HET versus STA) explained only 0.3% of the variation in the ‘dance around the means’ in the forest plots. On average, 76.2 ± 7.4% (mean ± standard deviation) of the study estimates for outcome measures from the HET cohorts fell within the 95% confidence interval of the meta-analytic mean estimate, compared to 73.8 ± 9.5% from the STA cohorts, providing no evidence for a higher coverage probability of estimates from HET cohorts compared to STA cohorts.

## Discussion

The ‘replicability crisis’ in biomedical research calls for effective solutions^7,15^. Similar to multi-center trials in clinical research, multi-laboratory studies might be an ideal solution for preclinical animal studies, but their implementation can be challenging due to logistical demands and intellectual property concerns^10,14^. Effective heterogenization of study samples within single-laboratory studies could potentially be an alternative approach, mimicking the benefits of multi-laboratory studies without the logistical and intellectual property challenges^9^.

Effective heterogenization of study populations requires the systematic variation of genetic and/or environmental factors that typically vary between independent replicate studies, thereby contributing to between-laboratory variation (that is, heterogeneity in meta-analyses) and thus poor replicability. Here we systematically tested whether heterogenization of study populations by including animals from different breeding sites is effective in improving the replicability of findings from single-laboratory animal studies.

The rationale behind choosing breeding sites as a heterogenization factor was based on our recent findings that common environmental differences between animal facilities can induce facility-specific phenotypes in mice^14^. Additionally, we considered the well-documented phenotypic variation that naturally occurs between different substrains of C57BL/6J mice^16–18^. Therefore, we expected the inclusion of mice from different breeding sites in study populations of single-laboratory studies to increase variation in many phenotypic traits, thereby mimicking the phenotypic variation that typically exists between different independent studies.

Contrary to our expectations, heterogenization of study populations by breeding site did not reduce between-laboratory variability compared to the conventional STA design. Several reasons may explain these unexpected findings. The main reason may be that breeding site contributed only little to total phenotypic variation, much less than test laboratory. As a result, there was little scope for heterogenization by breeding site to reduce between-laboratory variation. Given our previous findings that common environmental differences between animal-rearing facilities can induce persistent phenotypic differences from the molecular to the behavioral level in mice^14^, this finding was unexpected. One explanation could be that the rearing conditions in the facilities of professional breeders are much more similar (that is, STA) than the animal facilities of independent research institutions. Furthermore, the six breeding sites belonged to only three breeding companies. Thus, strictly STA operating procedures maintained across different breeding sites within companies could have further reduced phenotypic variation between mice from different breeding sites. Alternatively, the diversity of the mice within breeding sites may have been greater than expected, thereby limiting the scope for variation between breeding sites. This could, for example, be due to variation in age (the age of mice may vary by several days) and origin from different colony rooms.

The pair-housing of male mice could be another factor potentially contributing to larger diversity among mice from the same breeding site. Pair-housing may often result in despotic hierarchies among male mice, and it was found that circulating testosterone levels can differ by up to fivefold between dominant and subordinate males^19^. Such social effects may lead to substantial variability among mice of the same age and strain housed under identical conditions^20^. This may have been further corroborated by the need to single-house some animals for some time before testing due to escalating aggression. Although we accounted for this statistically, it remains possible that the biological effect was more pronounced^21,22^.

Importantly, the effect of the test laboratory on phenotypic variability was considerably stronger than that of the breeding site. Previous studies^23–25^ have indicated that the experimenter can have a strong influence on study outcomes, particularly emphasizing the impact of the experimenter’s biological sex on behavioral outcomes in rodents. Despite deliberately harmonizing test procedures and equipment and having the same female experimenter conduct all test procedures in all six laboratories, the test laboratory still contributed substantially to the total variation in outcome variables. This suggests that other factors of housing and husbandry that varied between test laboratories (for example, cage ventilation, cage types, environmental enrichment and animal care) must have influenced outcome variables. Thus, laboratory-specific microenvironments may have shaped the phenotypic states of the mice, thereby influencing the study outcomes. Such effects of the test laboratory would normally be even stronger, as the test equipment and test procedures that were standardized in this study would normally vary between test laboratories^3,13,14^.

In conclusion, we found no evidence that using mice from different breeding sites is potent enough to account for the variation that normally exists between results obtained in different laboratories. Although we here present a ‘negative finding’ or ‘null-result’, we believe that our study can serve as an example of how to implement heterogenization and how to assess the effectiveness of such an intervention on the external validity and replicability of experimental results. Our findings demonstrate substantial between-laboratory variation despite harmonized procedures, highlighting the need to strengthen our efforts to find practicable ways of heterogenizing study populations effectively to improve the replicability of results from basic and preclinical animal research.

## Methods

### Ethical statement

All animal experiments were conducted in full compliance with the Swiss Animal Welfare Ordinance (TSchV 455.1) and were approved by the Cantonal Veterinary Office in Bern, Switzerland (permit number BE88/20).

### Animal subjects and study design

In this multi-laboratory study, we focused on the C57BL/6J strain, as it is the most widely used strain in biomedical research^26–28^. As this was a proof-of-principle study, and to keep the study manageable, only male subjects were used. We selected male mice on the basis of our recent work, which demonstrated more pronounced phenotypic differences in C57BL/6J males raised in different facilities^14^.

In this study, we investigated the effectiveness of using animals from multiple breeding sites to introduce genetic and environmental variation as a solution to systematically increase variation within a single test laboratory and, consequently, decrease variation between test laboratories.

Mice were obtained from the following six commercial breeding sites (Supplementary Fig. 2):

i. Charles River Laboratories DE, Sulzfeld, Germany (B1; C57BL/6JCrl mice);
ii. Charles River Laboratories FRA, L’Arbresle, France (B2; C57BL/6JCrl mice);
iii. Charles River Laboratories UK, Kent, United Kingdom (B3; C57BL/6JCrl mice);
iv. Envigo RMS, Gannat, France (B4; C57BL/6JOlaHsd mice);
v. Envigo RMS, Gannat, France (B5; C57BL/6JRccHsd mice);
vi. Janvier Labs, Le Genest-Saint-Isle, France (B6; C57BL/6JRj mice).

The test laboratories were located at the following institutions:

i. Institute of Anatomy, University of Zürich (LAB 1);
ii. Division of Animal Welfare, Vetsuisse Faculty, University of Bern (LAB 2 and LAB 4);
iii. Central Animal Facilities, Experimental Animal Center, University of Bern (LAB 3);
iv. Institute of Veterinary Pharmacology and Toxicology, Vetsuisse Faculty, University of Zürich (LAB 5);
v. Laboratory of Neuroepigenetics, Brain Research Institute, University of Zurich and Institute for Neuroscience, ETH Zurich (LAB 6).

Each test laboratory provided space for animal housing, a test room for behavioral testing and an experimental room for tissue collection. Animal care was provided by each laboratory’s animal care staff.

For the STA study design, each test laboratory ordered all mice in one cohort from one of six breeding sites (each laboratory from a different site). For the systematically HET study design, each test laboratory received mice in proportionate numbers from five of the six breeding sites (excluding the one from which they ordered the mice for the STA study design). This resulted in a total of 12 replicate experiments (6 STA and 6 HET), in which 324 mice were used. The final number of mice used for STA and HET design in each test laboratory is presented in the data file.

As each test laboratory conducted the experiment independently, animals were delivered separately at an age of 12 weeks (*n* = 54 per test laboratory). The mice were shipped in groups of two cagemates in small or subdivided boxes. Due to the predisposition to elevated aggressive behavior in C57BL/6J males, the animals shipped together were housed together upon arrival.

Upon arrival, the animals were checked for health, then individually marked by fur cut, randomly assigned to cages by breeding site, and pair-housed under laboratory-specific housing and husbandry conditions (Supplementary Table 1) for 12 days before the onset of the test phase (Supplementary Fig. 3). Cage positions on the rack were also counterbalanced by breeding site (animal origin) and study design (STA or HET). Cages were cleaned 7 days after arrival and left undisturbed until the onset of the test phase to minimize disruption due to cage cleaning before testing. Food pellets and tap water were provided ad libitum. All mice were held under a constant 12-h light–dark cycle, but the time schedules differed between laboratories (Supplementary Table 1).

Since it has been shown that the test environment can have a profound influence on study outcomes^2,3,29^, the effects are very often attributed to the differences in test protocols (test time, equipment, illumination and so on)^30^ that normally exist between different laboratories. Thus, we controlled for all those factors by standardizing the test protocol and equipment across all six test laboratories. Additionally, studies have suggested that the experimenter performing the tests might have an effect on the outcome measures^23–25^, and that effect might be even stronger than the effect of the genotype on the same outcome measure^3^. In our experimental setup, we wanted to exclude that possibility, so the same experimenter (I.J.) performed behavioral testing and tissue collection in each test laboratory, thereby minimizing procedural variation that might affect outcome measures.

### Sample size calculation

The sample size for the HET study was partly determined by the requirement for a balanced study design within the HET cohorts. The sample size for the STA design was then incrementally adjusted until an estimated power of 0.8 was reached. To estimate the achieved power, we used simulated sampling. The R code for this simulation is attached as a supplementary file. In short, following simulated sampling with specific assumptions for the distribution of expected effect sizes, a principal component analysis was conducted over all 12 variables using orthogonal rotation, and the first principal component was taken as the input for an ANOVA analysis. The analysis aimed to determine how often the *f* ratios of the means squares for the HET and STA designs exceeded the threshold value of *f* = 6.6 (*P* ≤ 0.05 for 1 and 5 d.f.). The results showed that, under these assumptions, a significant main effect was found in 82.5% of the cases for a sample size of 24 animals in the STA cohort, indicating an achieved power of 0.825.

### Behavioral testing

To analyze phenotypic variation in behavior, we focused on changes in exploratory and emotional behavior by using one of the most common behavioral assay, the EPM^31,32^.

EPM testing was carried out in batches over two consecutive days during the dark phase, specifically between the first and fourth hours. The EPM apparatus was made of a gray-colored polycarbonate platform with a white removable floor. The platform comprised two opposite open arms (30 cm × 6 cm) and two opposite closed arms surrounded by 15-cm-high walls of the same dimensions. The central part that allows the animal to transit from arm to arm consists of a square with dimensions of 6 × 6 cm. The maze was elevated 40 cm above the ground, and the open arms were equipped with a small lip around the perimeter, 0.5 cm high, to ensure that no animals would fall off the maze. The illumination at the open arms was set to 140 lux.

Each test started by taking the mouse from the home cage and placing it in the center part of the EPM, facing the closed arm. Mice were allowed to freely explore the maze for 5 min. Both cagemates were tested simultaneously using two identical apparatuses placed next to each other but visually separated. The test order was balanced across breeding sites and experimental designs and randomized using the random number generator of the Mathematica software (version 11; Wolfram Research) separately for each test laboratory. Between trials, the apparatuses were sprayed with water containing odorless detergent, rinsed two times with water, and dried with paper towels.

The total distance traveled, the time spent in the open arms, and the number of entries into the open arms were measured from video recordings using EthoVision XT software (version 11.5; Noldus). The criterion for arm entry was when the center point of the animal (as detected by Ethovision) was in the arm.

### Tissue sampling procedure

Two days after the EPM test, animals were weighed and deeply anesthetized with an overdose of pentobarbital diluted in 0.9% saline (150 mg/kg, Esconarkon, Streuli Pharma AG). To avoid possible influences of the circadian rhythm on the blood clinical parameters, the procedures were performed during the first four hours of the light phase. The order of trials corresponds to the one used for behavioral testing.

Approximately 600–800 µl of blood was collected by cardiac puncture and transferred into potassium ethylenediaminetetraacetic acid (EDTA)-coated tubes (Micro sample tube K3 EDTA, 1.6 mg EDTA/ml blood, Sarstedt). Immediately after the puncture, the blood samples were placed on ice, and the animals were decapitated. Adrenal glands were removed, dissected from fat, and weighed using a precision scale (Mettler AE160, Mettler-Toledo). Within 1 h, the blood samples were centrifuged for 10 min at 4,000*g* and 4 °C. Plasma samples were transferred to new, labeled microcentrifuge tubes and stored at −80 °C until assayed.

### Blood clinical chemistry

We focused on blood chemistry parameters since they provide a good overview of the metabolic state and organ functions, as well as electrolyte and mineral homeostasis^33^.

All analyses were performed on a Roche Cobas c501 analyzer (Roche Diagnostics (Schweiz) AG). Total protein, albumin, globulin, creatinine, triglycerides and glucose, as well as the enzymatic activity of alanine transaminase and aspartate aminotransferase, were quantified photometrically with reagents provided by Roche Diagnostics. All procedures were performed according to the manufacturers’ protocols.

### Statistical analysis

Statistical analyses were performed in R (Supplementary Code)^34^. All analyses were performed for the same set of outcome variables: body weight at the day of sacrificing, relative adrenal weight, total distance traveled in the EPM, number of open arm entries in the EPM, time spent in the open arms in the EPM, and the blood plasma concentration of total protein, albumin, globulin, creatinine, enzymatic activity of alanine transaminase and aspartate aminotransferase, bilirubin, glucose and triglyceride.

To identify variance components attributable to breeder and laboratory, we first made a MANOVA with the outcome measures as dependent variables and laboratory, breeder and the interaction between laboratory and breeder as independent variables, followed by post-hoc mixed-effect regression models for each outcome variable with breeder, laboratory and their interaction as fixed factors and cage ID as a random factor. For calculating *P* values for the mixed-effect regression models, degrees of freedom were estimated using the Satterthwaite approximation.

Following the MANOVA, two separate LDAs were made: one with breeder as the response variable and the outcome variables as linear predictors, and one with laboratory as a response variable.

For comparing the variance of each outcome variable between STA and HET cohorts within each of the laboratory, we used Levene tests for equal variances with the significance threshold set to *α* = 0.05 (without correction for multiple testing). For combining outcome variables from the six laboratories to obtain a single measure for each outcome variable, we used Fisher’s method for combined probabilities^35^.

To investigate whether HET designs led to lower between-laboratory variation than STA designs, we ran for each outcome variable two separate mixed models with the outcome as a dependent variable, laboratory as fixed effect and cage ID as a random factor—one for the HET design and one for the STA design. We then compared the marginal *R*^2^ estimates. In a final step, we treated the results from the six laboratories as replicate studies and conducted random effect meta-analyses^36^ with the outcome as dependent variable and laboratory as random effect.

### Blinding

The experimenter performing weighing, the EPM test and tissue collection was blind to the ‘study design’, that is, STA or HET design. Blinding was done by two colleagues otherwise not involved in the execution of the experiments. Cages were assigned identification numbers so that the experimenter could not deduce the origin of the cages (that is, breeding site) from the ID number or the position of the cage. Blinding with regard to the test laboratory was not possible for weighing and organ collection since the experimenter needed to travel to each test facility. For the clinical chemistry analysis, the experimenter was blind to the study design and the test laboratory as well.

### Missing data and cases of single housing

During the experiment, a total of 16 mice were lost. In test laboratory 1, one mouse was euthanized immediately after arrival due to poor health conditions. The animal was apathetic and cold, and had wounds upon arrival. As a result, its cagemate was housed alone for the whole duration of the study. Additionally, in two cages, we observed an increased incidence of fighting, which resulted in small bite wounds. Consequently, a total of four animals had to be housed separately for a short period before behavioral testing.

In test laboratory 2, there was no need for single housing, and no animals were lost.

In test laboratory 3, three animals were euthanized in consultation with the responsible veterinarian due to a high level of wounding. This occurred 2 days before testing, resulting in the brief single housing of their cagemates.

In test laboratory 4, two cagemates were found dead during the habituation period; however, necropsy did not reveal a specific cause of death. Additionally, two more mice were euthanized due to high levels of injuries that occurred between two daily checks. Consequently, their cagemates were also single-housed. Furthermore, due to an observed incidence of aggression with tail bites, six additional mice from three cages were single-housed 2 days before testing.

In test laboratory 5, a total of five animals were lost. Cagemates from two cages, a total of four mice, were lost during the habituation period, while one animal was lost just before tissue collection, which did not result in the single housing of its cagemate. The necropsy of that animal showed the presence of cysts on both kidneys. Furthermore, 14 mice from seven cages needed to be individually housed due to incidences of aggression.

In test laboratory 6, one animal was euthanized immediately after arrival due to poor health conditions. Its cagemate was single-housed for the whole duration of the experiment. Additionally, two more animals were found dead in the home cage, 3 days before behavioral testing, which resulted in a period of single housing for their cagemates. Moreover, in three cages, a total of six mice needed to be single-housed 4 days before testing until the end of the experiment.

For the EPM testing, 25 data points were lost for each EPM outcome measure. Fourteen mice had their data lost due to animal euthanasia or death before testing, and an additional nine data points were lost due to technical problems during the transfer of recorded videos.

Two additional data points for blood clinical chemistry were excluded due to measurement errors.

Recalculating power given the final number of animals entering the analysis (299 for behavioral measures), there was a small drop in statistical power from 82.5% to 79.9% (Supplementary Material).

### Reporting summary

Further information on research design is available in the Nature Portfolio Reporting Summary linked to this article.

## Online content

Any methods, additional references, Nature Portfolio reporting summaries, source data, extended data, supplementary information, acknowledgements, peer review information; details of author contributions and competing interests; and statements of data and code availability are available at 10.1038/s41684-023-01307-w.

### Supplementary information

Supplementary Information Supplementary Figs. 1–3 and text (sample size calculation, simulated sampling and adjusted power analysis).

Reporting Summary

Supplementary Data A data file containing raw data for all tables and figures. This file serves as the foundation for all analyses.

Supplementary Code A file containing R code used for all analyses.

Supplementary Table 1 Laboratory-specific housing and husbandry conditions for each laboratory.

## Figures and Tables

**Extended Data Table 1:**
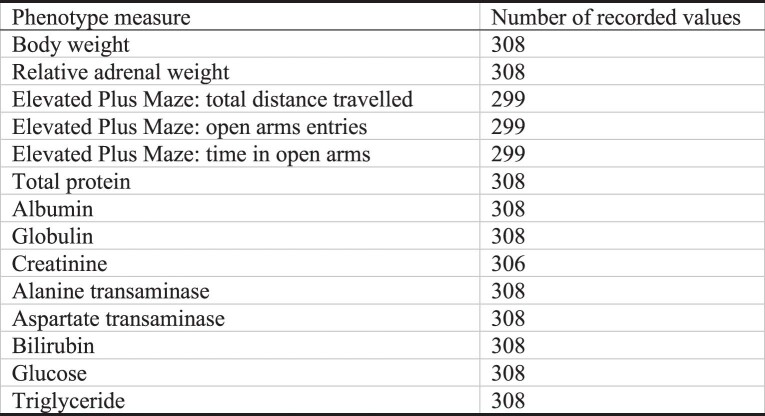
Number of recorded values per outcome measure

**Extended Data Table 2:**
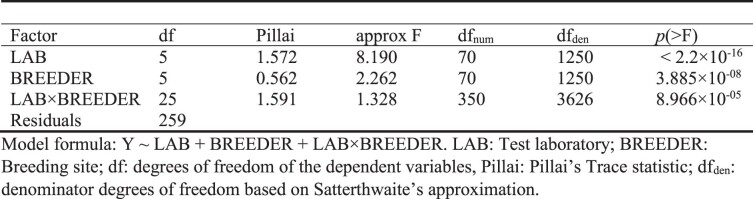
MANOVA outcome

**Extended Data Table 3:**
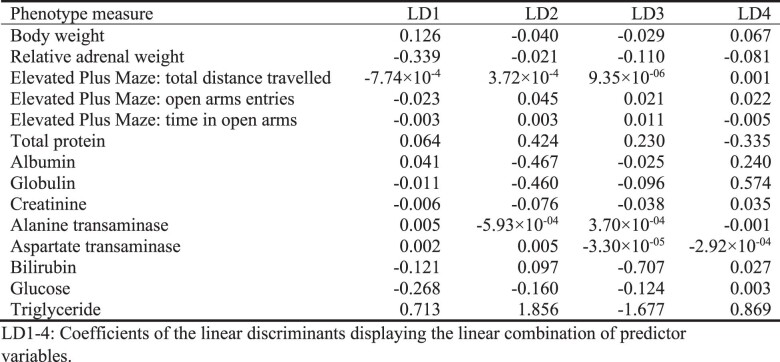
Linear discriminant function analysis for breeding site

**Extended Data Table 4:**
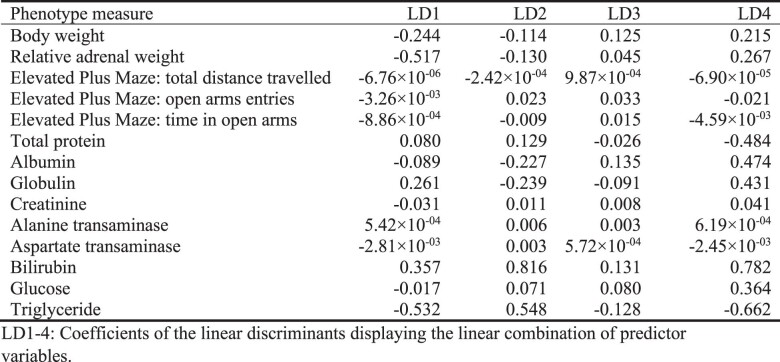
Linear discriminant function analysis for laboratory

**Extended Data Table 5:**
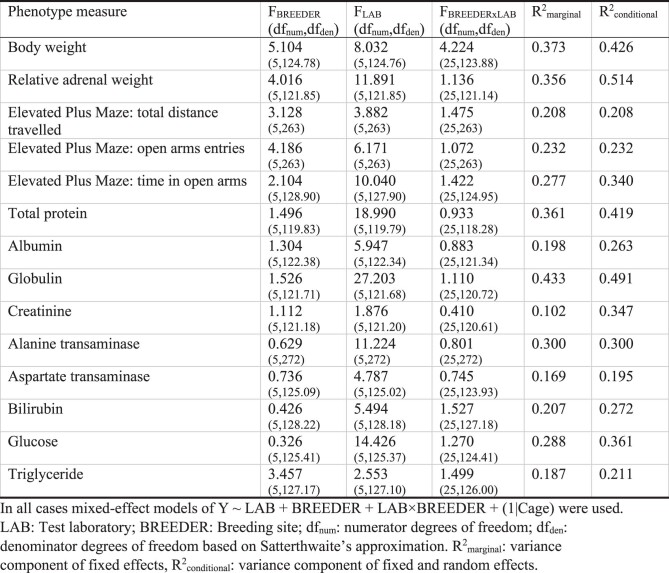
Outcomes for post-hoc type III ANOVAs

**Extended Data Table 6:**
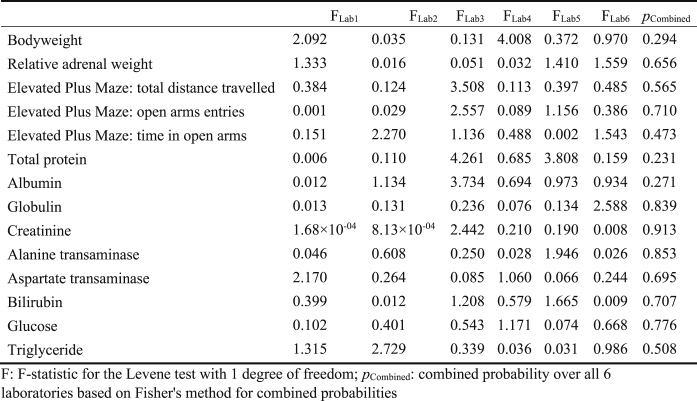
Levene Tests for equal variances

**Extended Data Table 7:**
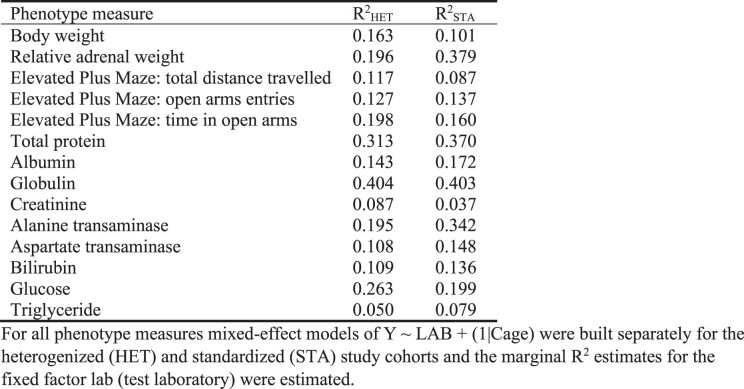
Variance explained by the factor Laboratory

## Data Availability

All data supporting the findings of this study together with the code are available within the article and its supplementary files (Supplementary Data and Supplementary Code).
