# Supplementary material for: Using mice from different breeding sites fails to improve replicability of results from single-laboratory studies
Source: Lab Anim (NY). 2023 Dec 27;53(1):18–22. doi: 10.1038/s41684-023-01307-w (PMC10766513; doi:10.1038/s41684-023-01307-w)
Supplement: Supplementary file 1 — Supplementary Figs. 1–3 and text (sample size calculation, simulated sampling and adjusted power analysis). [file 41684_2023_1307_MOESM1_ESM.pdf]

lab animal

https://doi.org/10.1038/s41684-023-01307-w

Using mice from different breeding sites fails to improve replicability of results from single-laboratory studies

In the format provided by the authors and unedited

Supplementary Information

Using mice from different breeding sites fails to improve replicability of results from single-laboratory studies

Ivana Jaric[1]*, Bernhard Voelkl[1], Irmgard Amrein[2], David P. Wolfer[2,3], Janja Novak[1], Carlotta Detotto[4], Ulrike Weber-Stadlbauer[5], Urs Meyer[5], Francesca Manuella [6, 7], Isabelle M Mansuy [6, 7] and Hanno Würbel[1]*

[1] Animal Welfare Division, Vetsuisse Faculty, University of Bern, Bern, Switzerland.

[2] Institute of Anatomy, Division of Functional Neuroanatomy, University of Zürich, Zürich, Switzerland.

[3] Department of Health Sciences and Technology, ETH Zürich, Zürich, Switzerland.

[4] Central Animal Facilities, Experimental Animal Center, University of Bern, Bern, Switzerland

[5] Institute of Pharmacology and Toxicology, Vetsuisse Faculty and Center of Neuroscience Zurich, University of Zurich, Zurich, Switzerland.

[6] Laboratory of Neuroepigenetics, Brain Research Institute, University of Zurich, Zurich, Switzerland.

[7] Institute for Neuroscience, Department of Health Sciences and Technology, ETH Zurich, Zurich, Switzerland.

* hanno.wuerbel@unibe.ch
* ivana.jaric@unibe.ch

Supplementary Figure 1: Raw data plotted for each outcome measure and test laboratory (LAB) for both STA and HET study designs

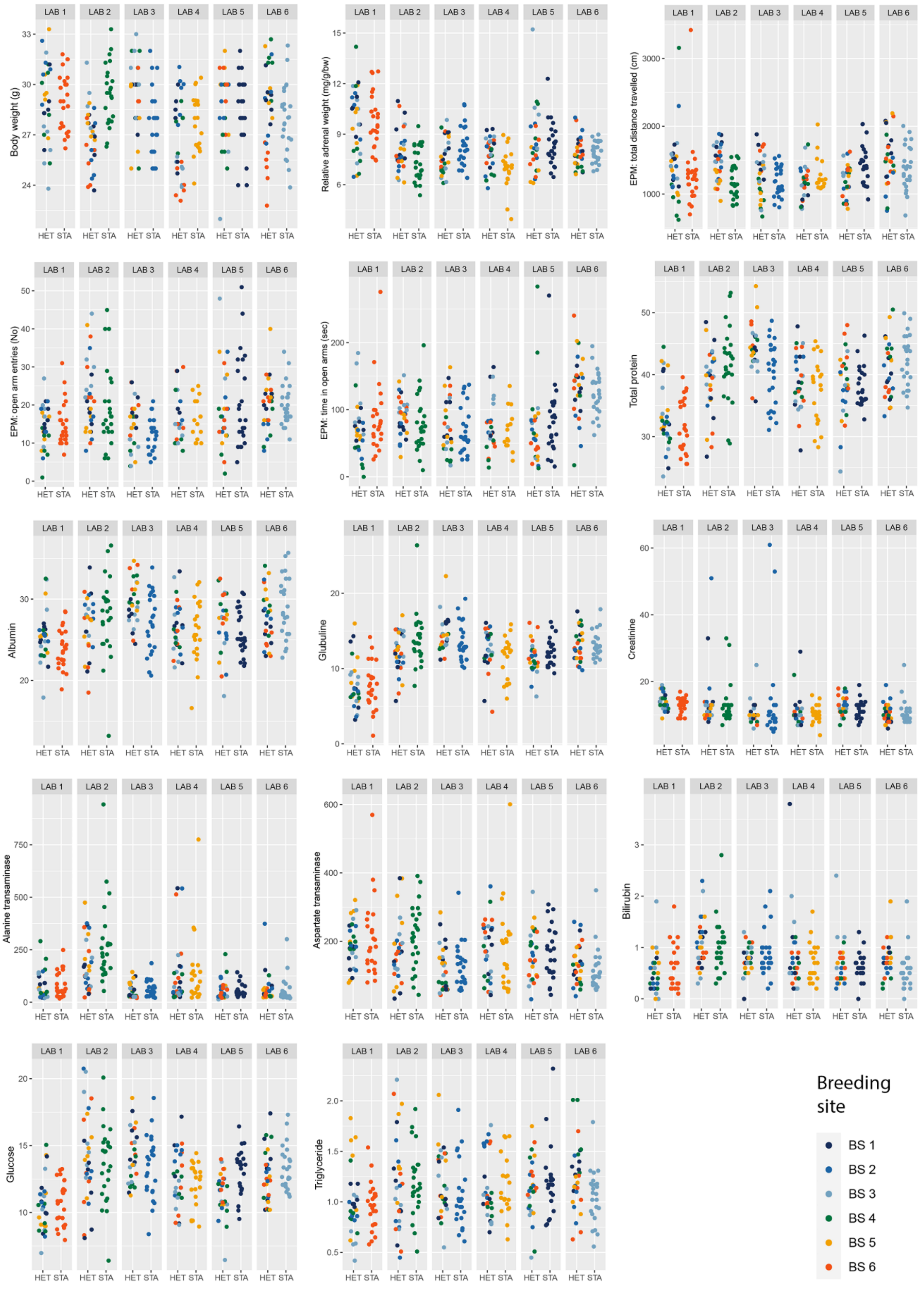

STA: standardized study design, HET: heterogenized study design, LAB: test laboratory, BS: breeding site.

Supplementary Figure 2: The origin of B57BL/6J mice we used in our experimental setup as heterogenization factor.

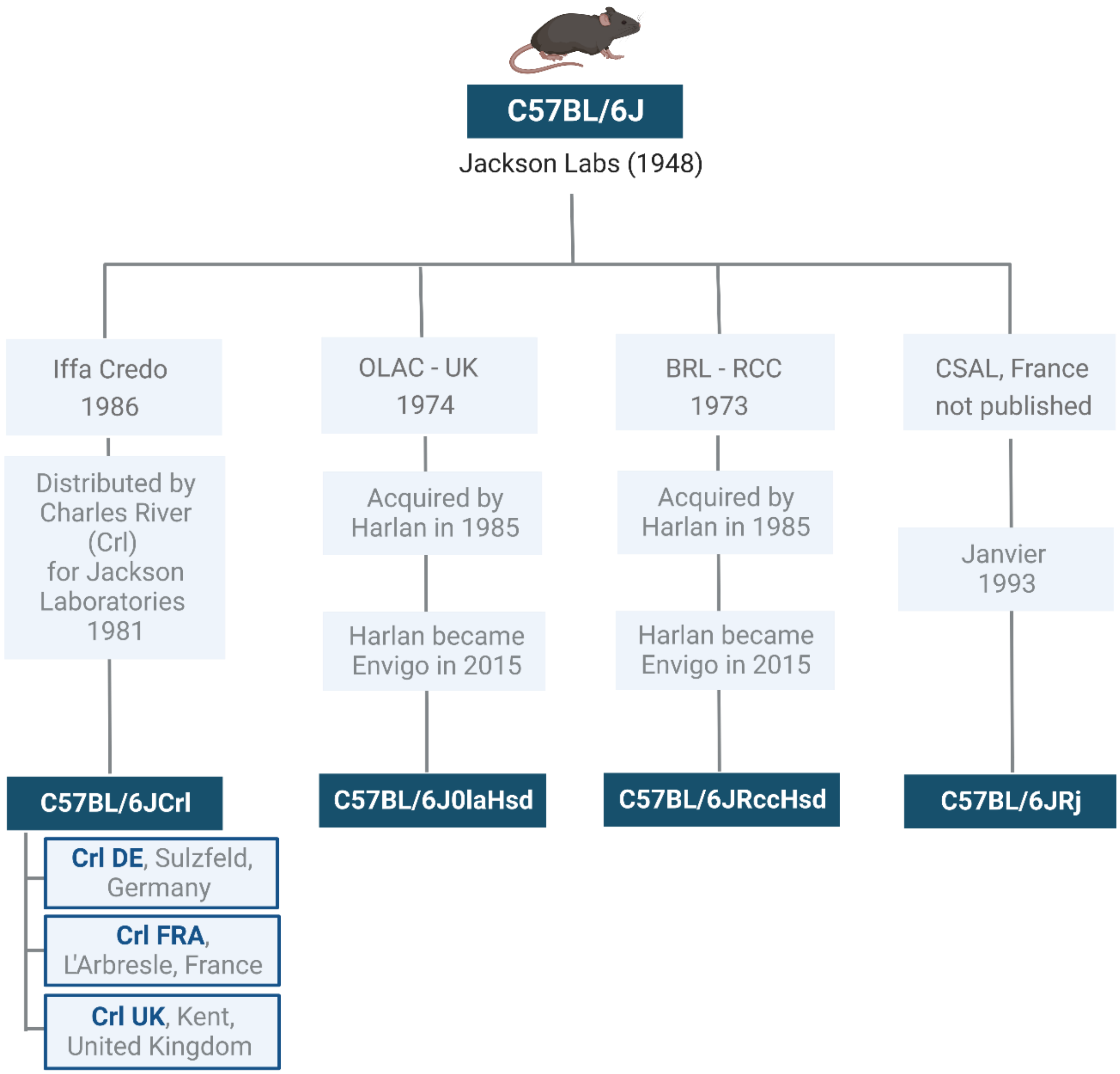

Supplementary Figure 3: Timeline of the study

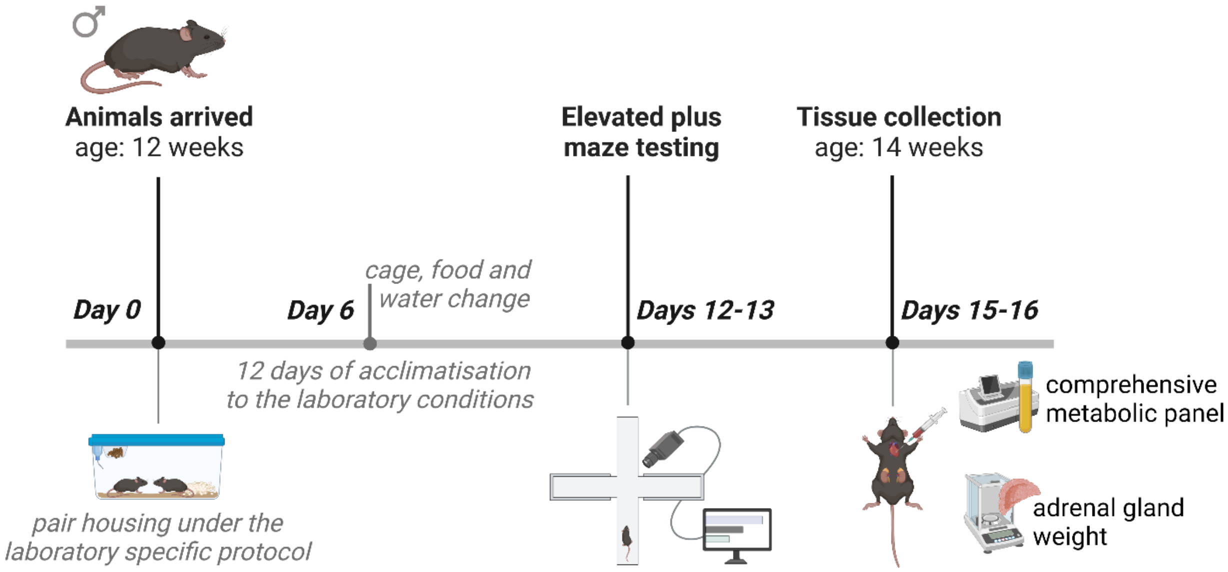

Supplementary Text:

Sample size calculation, Simulated Sampling and Adjusted power analysis with the R markdown code

The aim of the simulation was to get an estimate for the frequency with which we can find a difference in the variances between the STA and HET experimental design. Sample size are calculated for the case of 6 participating test laboratories. We assume 12 independent variables. For two third (8) variables we assume a feeble effect (sd of the latent variable = 0.1) of testing laboratory, for two variables we assume a medium sized effect (sd=2) and for two variables we assume a strong effect (sd=15); for the residual variation we assume a strong individual effect (sd=15). The critical statistic is the f-ratio of the means squares of the ANOVAs for the factor "lab" of the STA and HET groups with 1 and 5 df. For the HET group we set the sample size to 30 per laboratory, which allows a complete balanced design (with regard to rearing lab and breeder) within each STA group. For the STA group we started with a sample size of 30 per testing laboratory. As this resulted in a power of 0.884, which was above the aspired threshold level of 0.8, we subsequently reduced the sample size for the STA group in increments of two until the estimated power fell below 0.8. The recommended sample size for the STA group is the smallest sample size with an estimated power above 0.8, which is 24 subjects per group (*i.e.* testing laboratory) with an estimated power of 0.825.

```
#Initialization

setwd("M:/REP FAIL/Experiment B4")

library(lmerTest)

# Functions

simhom<- function(sdlab, sderr,n){ labeffect<-
    rnorm(6, mean=0, sd=sdlab) test<-c()

    for(k in 1:6){

        test<-c(test,rep(labeffect[k],n)+rnorm(n, mean=100, sd=sderr))

    }

    test

}

simhet<- function(sdlab, sderr){ labeffect<-rnorm(6,
    mean=0, sd=sdlab) test<-c()

    for(k in 1:6){
```

```
      labtest<-c(labtest,rep(labeffect[i],6)+rnorm(6, mean=100, sd=sderr))
    }
    pos<-c(6*k-5,6*k-4,6*k-3,6*k-2,6*k-1,6*k)
    test<-c(test,labtest[-pos])
  }
  test
}

# Simulation N(hom=30)
n=30
labhom<-as.factor(rep(1:6,each=n))
labhet<-as.factor(rep(1:6,each=30))
set.seed(1066)

f1<-c()
for(i in 1:1000){
  outcomes<-mapply(simhom,sample(c(rep(0.1,8),rep(1,2),rep(15,2))),rep(15,12)
,n)
  pca<-prcomp(outcomes)
  model<-lm(pca$x[,1]~labhom)
  atab<-anova(model)
  ms1<-atab$`Mean Sq`[1]
  outcomes<-mapply(simhet,sample(c(rep(0.1,8),rep(1,2),rep(15,2))),rep(15,12)
)
  pca<-prcomp(outcomes)
  model<-lm(pca$x[,1]~labhet)
  atab<-anova(model)
  ms2<-atab$`Mean Sq`[1]
  f1<-c(f1,ms1/ms2)
}
length(which(f1 >6.6))/1000

## [1] 0.884 power estimate

# Simulation N(hom=24)
n=24
labhom<-as.factor(rep(1:6,each=n))
labhet<-as.factor(rep(1:6,each=30))
set.seed(1066)

f1<-c()
for(i in 1:1000){
  outcomes<-mapply(simhom,sample(c(rep(0.1,8),rep(1,2),rep(15,2))),rep(15,12)
,n)
  pca<-prcomp(outcomes)
  model<-lm(pca$x[,1]~labhom)
  atab<-anova(model)
  ms1<-atab$`Mean Sq`[1]
  outcomes<-mapply(simhet,sample(c(rep(0.1,8),rep(1,2),rep(15,2))),rep(15,12)
)
```

```
  pca<-prcomp(outcomes)
  model<-
  lm(pca$x[,1]~labhet)
  atab<-anova(model)
  ms2<-atab$`Mean
  Sq`[1] f1<-
  c(f1,ms1/ms2)
}
length(which(f1 >6.6))/1000

## [1] 0.825 power estimate

# Simulation N(hom=22)
n=22
labhom<-
as.factor(rep(1:6,each=n))
labhet<-
as.factor(rep(1:6,each=30))
set.seed(1066)

f1<-c()
for(i in 1:1000){
  outcomes<-
  mapply(simhom,sample(c(rep(0.1,8),rep(1,2),rep(15,2))),rep(15,12)
,n)
  pca<-prcomp(outcomes)
  model<-
  lm(pca$x[,1]~labhom)
  atab<-anova(model)
  ms1<-atab$`Mean Sq`[1]
  outcomes<-
  mapply(simhet,sample(c(rep(0.1,8),rep(1,2),rep(15,2))),rep(15,12)
)
  pca<-prcomp(outcomes)
  model<-
  lm(pca$x[,1]~labhet)
  atab<-anova(model)
  ms2<-atab$`Mean
  Sq`[1] f1<-
  c(f1,ms1/ms2)
}
length(which(f1 >6.6))/1000

## [1] 0.757 power estimate
```

Adjusted power given the final sample size

The aim of the simulation is to get an estimate for the "potentially achieved power" given the observed attrition and missing data (minimal sample: n=299 for behaviour: Standardized: 134, Heterogenized: 165).

```
#Initialization

setwd("F:/REP FAIL/Experiment B4")

library(lmerTest)

## Warning: package 'lmerTest' was built under R version 3.6.3

## Loading required package: lme4

## Loading required package: Matrix

##
## Attaching package: 'lmerTest'

## The following object is masked from 'package:lme4':
##
##     lmer

## The following object is masked from 'package:stats':
##
##     step

# Functions

simhom<- function(sdlab, sderr,n){
  labeffect<-rnorm(6, mean=0, sd=sdlab)
  test<-c()
  for(k in 1:6){
    test<-c(test,rep(labeffect[k],n)+rnorm(n, mean=100, sd=sderr))
  }
  test
}

simhet<- function(sdlab, sderr){
  labeffect<-rnorm(6, mean=0, sd=sdlab)
  test<-c()
  for(k in 1:6){
    labtest<-c()
    for(i in 1:6){
      labtest<-c(labtest,rep(labeffect[i],6)+rnorm(6, mean=100, sd=sderr))
    }
    pos<-c(6*k-5,6*k-4,6*k-3,6*k-2,6*k-1,6*k)
    test<-c(test,labtest[-pos])
  }
  test
}

# Standardized: "Hom": 134, Heterogenized "Het": 165
```

```
n=24
labhom<-as.factor(rep(1:6,each=n))
labhet<-as.factor(rep(1:6,each=30))
set.seed(1066)

f1<-c()
for(i in 1:1000){
  outcomes<-mapply(simhom,sample(c(rep(0.1,8),rep(1,2),rep(15,2))),rep(15,
12),n)
  sqhom<- sort(sample(seq(1:144),134))
  pca<-prcomp(outcomes[sqhom,])
  tempdf<-data.frame(pca$x[,1],labhom[sqhom])
  names(tempdf)<-c("pc1","lab")
  model<-lm(pc1~lab, data=tempdf)
  atab<-anova(model)
  ms1<-atab$`Mean Sq`[1]
  outcomes<-mapply(simhet,sample(c(rep(0.1,8),rep(1,2),rep(15,2))),rep(15,
12))
  sqhet<- sort(sample(seq(1:180),165))
  pca<-prcomp(outcomes[sqhet,])
  tempdf<-data.frame(pca$x[,1],labhet[sqhet])
  names(tempdf)<-c("pc1","lab")
  model<-lm(pc1~lab, data=tempdf)
  atab<-anova(model)
  ms2<-atab$`Mean Sq`[1]
  f1<-c(f1,ms1/ms2)
}
length(which(f1 >6.6))/1000

## [1] 0.799
```

Downsampling Standardized and Heterogenized cohorts to 134 and 165 animals respectively gives a slightly lower power (original power estimate for 324 animals was 0.825).
