# Supplementary material for: Using mice from different breeding sites fails to improve replicability of results from single-laboratory studies
Source: Lab Anim (NY). 2023 Dec 27;53(1):18–22. doi: 10.1038/s41684-023-01307-w (PMC10766513; doi:10.1038/s41684-023-01307-w)
Supplement: Supplementary file 2 — Reporting Summary [file 41684_2023_1307_MOESM2_ESM.pdf]

nature portfolio

Corresponding author(s): Ivana Jaric; ivana.jaric@unibe.ch
Hanno Würbel; hanno.wuerbel@unibe.ch

Last updated by author(s): Nov 14, 2023

Reporting Summary

Nature Portfolio wishes to improve the reproducibility of the work that we publish. This form provides structure for consistency and transparency in reporting. For further information on Nature Portfolio policies, see our Editorial Policies and the Editorial Policy Checklist.

Statistics

For all statistical analyses, confirm that the following items are present in the figure legend, table legend, main text, or Methods section.

n/a	Confirmed	
☐	☒	The exact sample size (n) for each experimental group/condition, given as a discrete number and unit of measurement
☐	☒	A statement on whether measurements were taken from distinct samples or whether the same sample was measured repeatedly
☐	☒	The statistical test(s) used AND whether they are one- or two-sided<br>*Only common tests should be described solely by name; describe more complex techniques in the Methods section.*
☒	☐	A description of all covariates tested
☐	☒	A description of any assumptions or corrections, such as tests of normality and adjustment for multiple comparisons
☐	☒	A full description of the statistical parameters including central tendency (e.g. means) or other basic estimates (e.g. regression coefficient) AND variation (e.g. standard deviation) or associated estimates of uncertainty (e.g. confidence intervals)
☐	☒	For null hypothesis testing, the test statistic (e.g. F, t, r) with confidence intervals, effect sizes, degrees of freedom and P value noted<br>*Give P values as exact values whenever suitable.*
☒	☐	For Bayesian analysis, information on the choice of priors and Markov chain Monte Carlo settings
☐	☒	For hierarchical and complex designs, identification of the appropriate level for tests and full reporting of outcomes
☐	☒	Estimates of effect sizes (e.g. Cohen's d, Pearson's r), indicating how they were calculated

Our web collection on statistics for biologists contains articles on many of the points above.

Software and code

Policy information about availability of computer code

Data collection	Behavioral data were collected from video recordings using EthoVision XT software (version 11.5; Noldus, Wageningen, the Netherlands). Adrenal glands data were generated by using a precision scale (Mettler AE160, Mettler-Toledo, Switzerland). Blood clinical chemistry analyses were performed on a Roche Cobas c501 analyzer (Roche Diagnostics (Schweiz) AG, Rotkreuz, Switzerland).
Data analysis	Statistical analyses are performed in R and script is provided as supplementary code and sours data files.

For manuscripts utilizing custom algorithms or software that are central to the research but not yet described in published literature, software must be made available to editors and reviewers. We strongly encourage code deposition in a community repository (e.g. GitHub). See the Nature Portfolio guidelines for submitting code & software for further information.

Data

Policy information about availability of data

All manuscripts must include a data availability statement. This statement should provide the following information, where applicable:

- Accession codes, unique identifiers, or web links for publicly available datasets
- A description of any restrictions on data availability
- For clinical datasets or third party data, please ensure that the statement adheres to our policy

Provide your data availability statement here.

Human research participants

Policy information about studies involving human research participants and Sex and Gender in Research.

Reporting on sex and gender	NA
Population characteristics	NA
Recruitment	NA
Ethics oversight	NA

Note that full information on the approval of the study protocol must also be provided in the manuscript.

Field-specific reporting

Please select the one below that is the best fit for your research. If you are not sure, read the appropriate sections before making your selection.

☒ Life sciences ☐ Behavioural & social sciences ☐ Ecological, evolutionary & environmental sciences

For a reference copy of the document with all sections, see nature.com/documents/nr-reporting-summary-flat.pdf

Life sciences study design

All studies must disclose on these points even when the disclosure is negative.

Sample size	The sample size for the heterogenized (HET) study was partly determined by the requirement for a balanced study design within the HET cohorts. The sample size for the standardized (STA) design was then incrementally adjusted until an estimated power of 0.8 was reached. In order to estimate the achieved power, we used simulated sampling. The R-code for this simulation is attached as a supplementary file. In short, following simulated sampling with specific assumptions for the distribution of expected effect sizes, a principal component analysis was conducted over all 12 variables using orthogonal rotation, and the first principal component was taken as the input for an ANOVA analysis. The analysis aimed to determine how often the f-ratios of the means squares for the HET and STA designs exceeded the threshold value of f=6.6 (p<=0.05 for 1 and 5 df). The results showed that under these assumptions, a significant main effect was found in 82.5% of the cases for a sample size of 24 animals in the STA cohort, indicating an achieved power of 0.825.
Data exclusions	During the experiment, a total of 16 mice were lost. In testing laboratories 1 and 6, two mice were euthanized immediately after arrival due to poor health conditions. In testing laboratory 4, two mice were found dead during the habituation period, while in testing laboratories 3 and 5, two mice were found dead just before the final tissue collection. However, necropsy did not reveal a specific cause of death.<br>Additionally, during the habituation period, 11 mice were euthanized due to high levels of wounding: two in testing laboratory 3, two in testing laboratory 4, four in testing laboratory 5, and two in testing laboratory 6. All euthanasia procedures were performed after consultation with the responsible veterinarians in each testing facility.<br>For the EPM testing, 25 data points were lost for each EPM outcome measure. Fourteen mice had their data lost due to animal euthanasia or death prior to testing, and an additional nine data points were lost due to technical problems during the transfer of recorded videos.<br>Two additional data points for blood clinical chemistry were excluded due to measurement error
Replication	Since this was animal study, we used biological replicates.
Randomization	The animals were randomly assigned to cages by breeding site. Cage positions on the rack were also counterbalanced by breeding site (animal origin) and study design (STA or HET).
Blinding	The experimenter performing weighing, EPM test and tissue collection was blind to the "study design", i.e STA or HET design. Blinding was done by two colleagues otherwise not involved in the execution of the experiments. Cages were assigned identification numbers so that the experimenter cannot deduce the origin of the cages (i.e. breeding site) from the ID number or the position of the cage. Blinding with regard to testing laboratory was not possible for weighing and organ collection since the experimenter needed to travel to each testing facility. For the clinical chemistry analysis, the experimenter was blind to the "study design" and the testing laboratory, as well.

Reporting for specific materials, systems and methods

We require information from authors about some types of materials, experimental systems and methods used in many studies. Here, indicate whether each material, system or method listed is relevant to your study. If you are not sure if a list item applies to your research, read the appropriate section before selecting a response.

Materials & experimental systems

n/a	Involved in the study
☒	☐ Antibodies
☒	☐ Eukaryotic cell lines
☒	☐ Palaeontology and archaeology
☐	☒ Animals and other organisms
☒	☐ Clinical data
☒	☐ Dual use research of concern

Methods

n/a	Involved in the study
☒	☐ ChIP-seq
☒	☐ Flow cytometry
☒	☐ MRI-based neuroimaging

Animals and other research organisms

Policy information about studies involving animals; ARRIVE guidelines recommended for reporting animal research, and Sex and Gender in Research

Laboratory animals	We focused on the C57BL/6J male mice (12 week old) which were obtained from multiple breeding sites to introduce genetic and environmental variation. Mice were obtained from the following six commercial breeding sites: i) Charles River Laboratories DE, Sulzfeld, Germany (B1; C57BL/6JCrl mice); ii) Charles River Laboratories FRA, L'Arbresle, France (B2; C57BL/6JCrl mice); iii) Charles River Laboratories UK, Kent, United Kingdom (B3; C57BL/6JCrl mice); iv) Envigo RMS, Gannat, France (B4; C57BL/6JOlaHsd mice); v) Envigo RMS, Gannat, France (B5; C57BL/6JRccHsd mice); vi) Janvier Labs, Le Genest-Saint-Isle, France (B6; C57BL/6JRj mice).
Wild animals	This study did not include wild-animals.
Reporting on sex	As this was a proof-of-principle study and to keep the study manageable, only male subjects were used. We selected male mice based on our recent work, which demonstrated more pronounced phenotypic differences in C57BL/6J males raised in different facilities (Jaric et al. 2022 doi.org/10.1371/journal.pbio.3001837).
Field-collected samples	This study did not include field-collected samples.
Ethics oversight	All animal experiments were conducted in full compliance with the Swiss Animal Welfare Ordinance (TSchV 455.1) and were approved by the Cantonal Veterinary Office in Bern, Switzerland (permit number: BE88/20).

Note that full information on the approval of the study protocol must also be provided in the manuscript.
